# Disparities in melanoma incidence and mortality in rural versus urban Michigan

**DOI:** 10.1002/cnr2.1713

**Published:** 2022-10-14

**Authors:** Richard A. Shellenberger, Timothy M. Johnson, Fatima Fayyaz, Bhanu Swamy, Jeremy Albright, Alan C. Geller

**Affiliations:** ^1^ St. Joseph Mercy Ann Arbor Hospital Ypsilanti Michigan USA; ^2^ Ganger Dermatology and Departments of Dermatology, Otolaryngology, and Surgery Michigan Medicine Ann Arbor Michigan USA; ^3^ Northwell Health Cancer Institute Zucker School of Medicine New Hyde Park New York USA; ^4^ Methods Consultants of Ann Arbor Ypsilanti Michigan USA; ^5^ Harvard TH Chen School of Public Health Cambridge Massachusetts USA

**Keywords:** cutaneous melanoma, melanoma incidence, melanoma‐specific mortality, rural disparities, rural healthcare

## Abstract

**Introduction:**

We sought to identifying the possible existence of disparities between rural and urban residents of Michigan for the incidence by stage of disease and disease‐specific mortality for cutaneous melanoma (CM).

**Methods:**

Incidence rates for stage of disease and disease‐specific mortality of cutaneous melanoma were calculated and controlled for gender, age, and area of residence from January 1, 2014, to December 31, 2018, from data collected form the Michigan Department of Health and Human Services and the Centers for Disease Control and Prevention.

**Results:**

The incidence rates for CM were significantly higher in rural Michigan counties, from 2014–2018, for all patients, both age groups, both genders and all stages. Melanoma‐specific mortality rates were also significantly higher for all patients, both age groups and both genders in rural Michigan counties. Using logistic regression analysis, while controlling for age and gender, rural Michigan counties continued to have a higher melanoma‐specific morality rate during our study period (OR = 1.491; 95% CI, 1.27–1.74; *p* = <.001).

**Conclusion:**

We found significant disparities in the incidence rates and disease specific mortality for cutaneous melanoma in rural compared to urban Michigan from 2014–2018.

## 1 INTRODUCTION

Sociodemographic factors have been associated with the incidence, stage of disease, treatment, trial involvement and prognosis for many cancers.^1^ Access to healthcare in the United States (US) is affected by income, education, travel distance, provider supply, and rurality. Although the terms rural and urban have been notoriously challenging to concisely define; US State of Michigan defines rural counties as those lacking any core urban area with a population of at least 50 000 persons.^2^, ^3^ The incidence rate and mortality rate of many cancers have been shown to be higher in rural populations compared to urban.^4^, ^5^, ^6^ For colorectal, breast, prostate, and cervical cancers; lower rates of preventative screening, higher rates of more advanced stage of disease being diagnosed and increased cancer associated mortality have also been associated with rurality.^4^, ^5^, ^6^ For cutaneous melanoma (CM) the stage of the initial diagnosis has shown to be negatively correlated with socioeconomic status and physician supply; however, very little data is available for rural disparities for this disease.^7^, ^8^ The purpose for our study was to further define possible rural disparities for CM.

## 2 METHODS

We obtained data for the incidence of CM and melanoma‐specific mortality by request from the Michigan Resident Cancer Incidence File, Michigan Department of Health and Human Services, Division of Vital Records and Health Statistics for patients residing in rural and urban counties from January 1, 2014, to December 31, 2018. Michigan has applied the definition of rural and urban by assigning a binary classification to each county having less or more than 50% of its population living in rural or urban areas as defined by the US Census Bureau.^2^, ^3^ Rural and urban counties were defined by using the 2013 Rural–Urban Continuum Codes, which categorize counties based upon their population and proximity to metropolitan areas.^9^ Data in the Michigan Resident Cancer Incidence File is reported for age group, gender, insurance status, and the Surveillance, Epidemiology and End Result (SEER) summary stage of disease. The SEER summary stages for CM include: localized (no signs of cancer beyond the primary skin site); regional (cancer which has spread to regional lymph nodes or in‐transit disease); distant (cancer which has spread to distant parts of the body, such as the lungs, liver, or skin on other parts of the body); and invasive unknown stages.^10^ Incidence and melanoma‐specific mortality for five racial groups were obtained from the US Cancer Statistics: Data Visualizations, Centers for Disease Control and Prevention (CDC) for all Michigan counties.^11^

**FIGURE 1:**
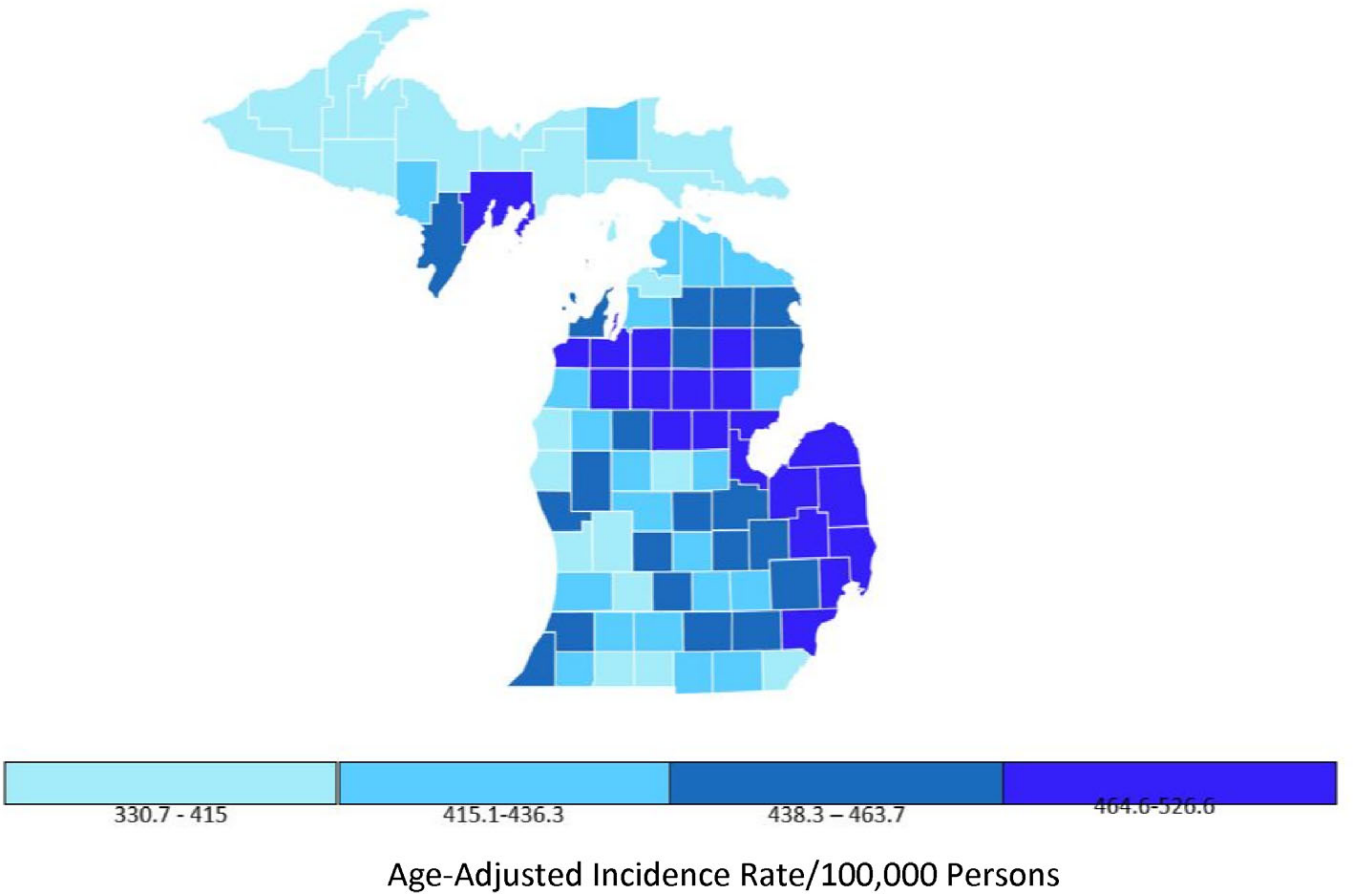
Rate of all types of new cancers in Michigan. All ages, all races and ethnicities, male and female, 2015–2018

**FIGURE 2:**
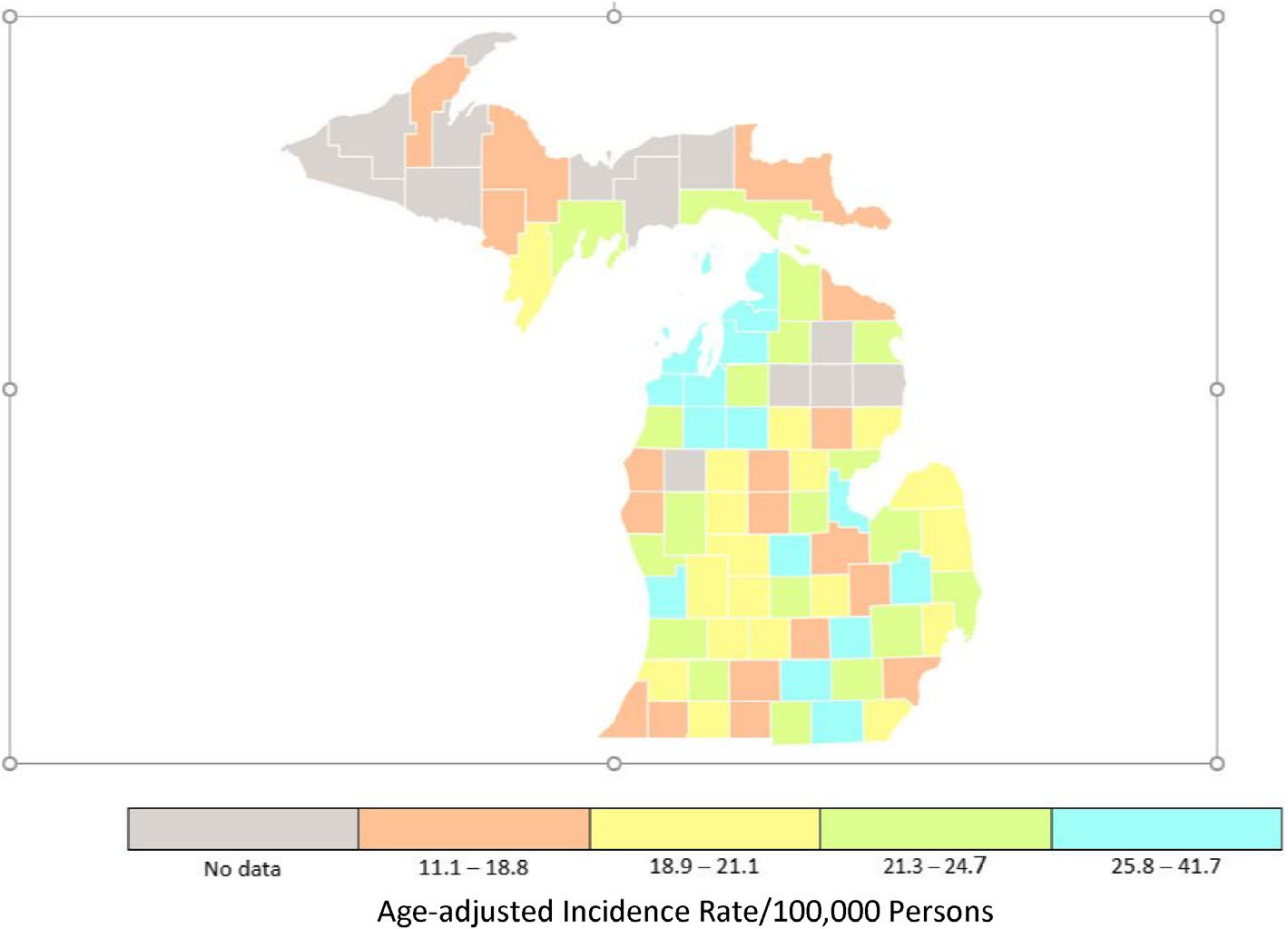
Rate of melanoma of the skin in Michigan. All ages, all races and ethnicities, male and female, 2015–2018

**FIGURE 3:**
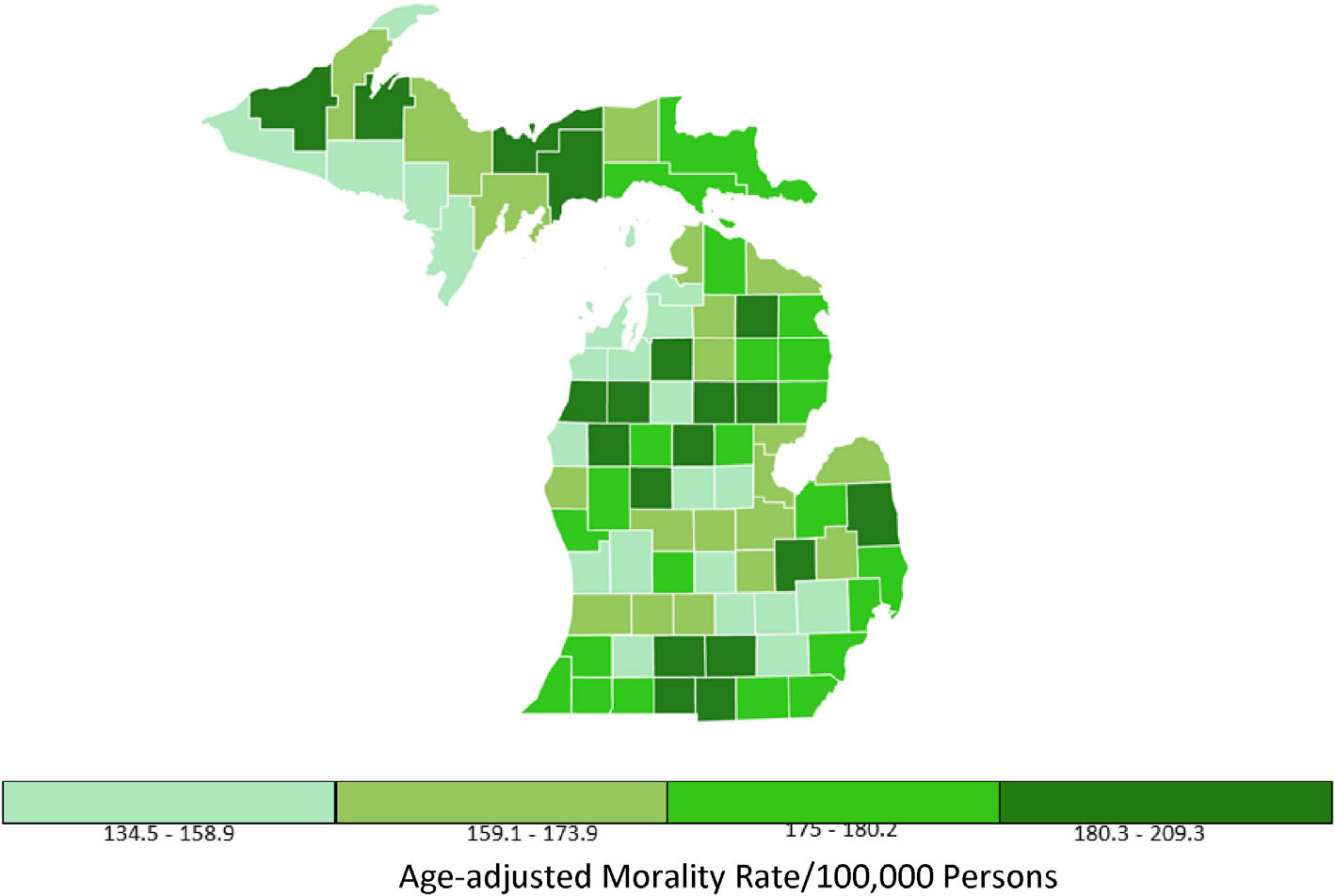
Rate of death for all cancer types in Michigan. All ages, all races and ethnicities, male and female, 2015–2018

**FIGURE 4:**
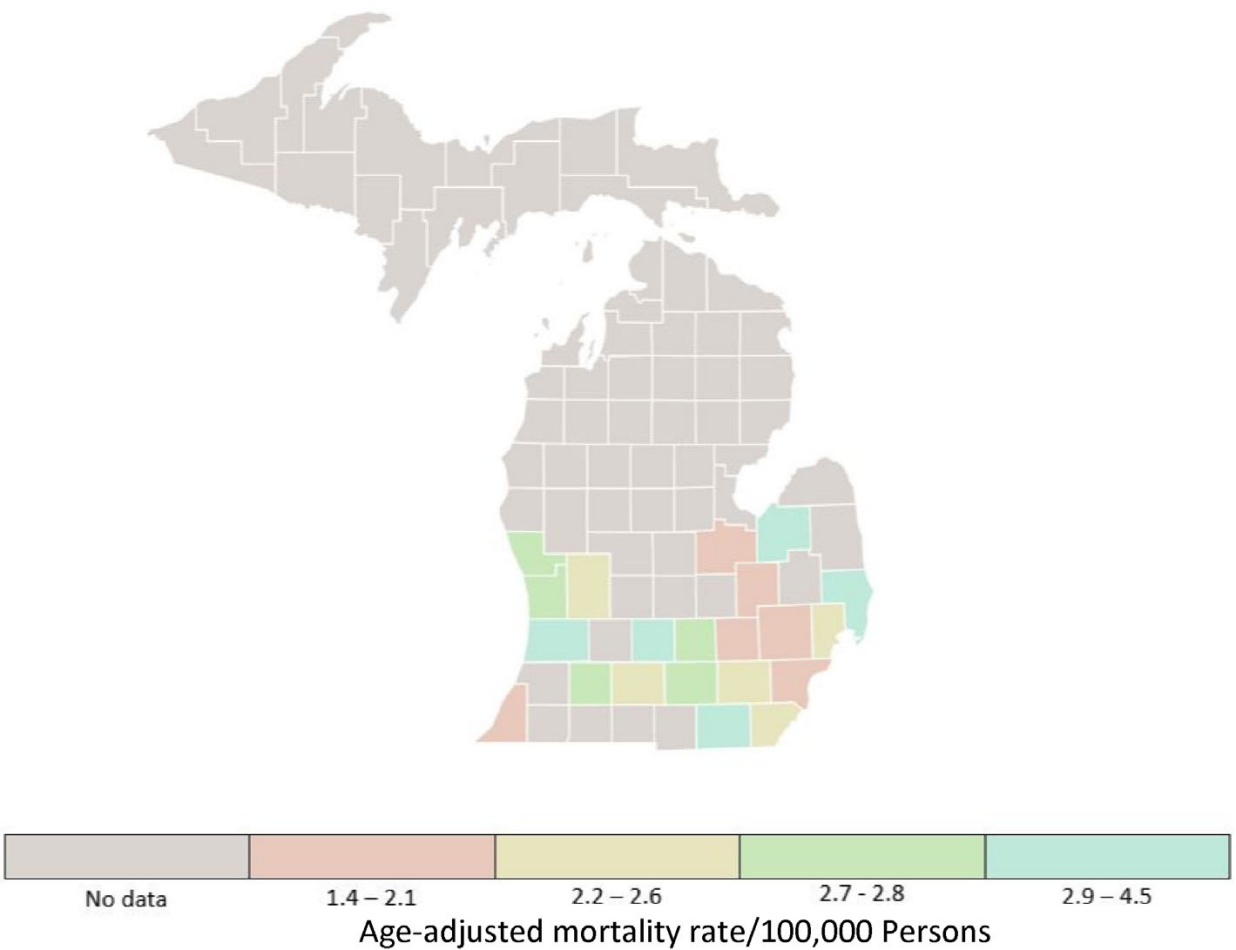
Rate of all deaths from melanoma of the skin. All ages, all races and ethnicities, male and female, 2014–2018

Information regarding the number of dermatologists practicing in urban and rural Michigan counties was retrieved through a physician finder from the American Academy of Dermatology and then verified with Google searches to ensure accuracy and completeness.^12^ Dermatologists practicing in more than one county were counted for each location. Quartile plots for incidence rates and mortality rates for all cancers and for melanoma of the skin in Michigan counties form 2014–2018 are presented (Figures 1, 2, 3, 4).^11^

### 2.1 Statistical analysis

Incidence rates and mortality rates were calculated using the direct method. All analysis was performed in R (Version 3.6.0). Logistic regression models were fit for the outcomes of melanoma incidence and disease specific mortality, adjusting for region (urban vs. rural), age (under 65 vs. 65 and over), and gender using aggregated datafiles for the state of Michigan pulled from the years 2014 to 2018. Rate ratios (RR) and 95% confidence intervals are presented.

### 2.2 Ethical approval

This study was deemed to be exempt for ethical approval by the institutional review board at Saint Joseph Mercy Ann Arbor Hospital, Ann Arbor, MI.

## 3 RESULTS

Results were obtained between January of 2021 and February of 2022. Rate ratios (RR) are reported for incidence rates and melanoma‐specific mortality rates for CM by stage of disease (Table 1). The incidence rates for both age groups, both genders, and all SEER summary stages were significantly higher for people residing in rural counties compared with urban counties (Table 1). Melanoma‐specific mortality rates were also significantly higher in rural Michigan counties for all patients combined as well as in both age groups and both genders studied.

**TABLE 1:** Incidence and melanoma‐specific mortality of cutaneous melanoma in rural and urban Michigan counties 2014–2018

	Incidence					Mortality				
	Rural		Urban			Rural		Urban		
	Cases	Rate/ 100 000)	Cases	Rate/100 000 per	Rate ratio [95% CI], *p*	Cases	Rate/100 000)	Case	Rate (per 100/000)	Rate ratio [95% CI], *p*
Total	2455	28.75	9135	22.06	1.30 [1.25, 1.36], *p* < .001	327	3.83	1025	2.48	1.55 [1.37, 1.75], *p* < .001
Age group										
<65 years old	1172	16.28	4834	13.43	1.21 [1.14, 1.29], *p* < .001	133	1.85	399	1.11	1.67 [1.37, 2.03], *p* < .001
65 years old and older	1283	95.70	4301	79.29	1.21 [1.13, 1.28], *p* < .001	194	14.47	626	11.54	1.25 [1.07, 1.47], *p* = .006
Sex										
Men	1435	34.3	5170	25.48	1.35 [1.27, 1.43], *p* < .001	232	5.54	664	3.27	1.69 [1.46, 1.97], *p* < .001
Women	1020	23.42	3965	18.78	1.25 [1.16, 1.29], *p* < .001	95	2.18	361	1.71	1.28 [1.02, 1.60], *p* = .034
SEER summary stage										
Localized	1850	21.66	7327	17.69	1.22 [1.16, 1.29], *p* < .001	X	X	X	X	
Regional	250	2.93	877	2.12	1.38 [1.20, 1.59], *p* < .001	X	X	X	X	
Distant	149	1.74	470	1.14	1.54 [1.28, 1.85], *p* < .001	X	X	X	X	
Invasive unknown	206	2.41	467	1.13	2.14 [1.82, 2.52], *p* < .001	X	X	X	X	

From 2014–2018, the results of the logistic regression model for melanoma‐specific mortality are presented in Table 2. We controlled for age, gender and area of residence for our logistic regression analysis. We were unable to use race as a covariate as data from the CDC was suppressed due to low numbers of CM in racial groups other than white in all rural Michigan counties. Controlling for gender and age, the risk of melanoma‐specific mortality was significantly higher for people residing in rural counties compared with urban counties (RR = 1.491; 95% CI, 1.27–1.74; *p* = <.001). Controlling for region and age, the risk of melanoma‐specific mortality is higher for males as compared to females (RR = 2.213; 95% CI, 1.94–2.52; *p* = .001). Controlling for region and gender, the melanoma‐specific mortality is significantly higher in persons aged 65 and older as compared to those younger than 65 (RR = 3.986; 95% CI, 3.52–4.53; *p* = <.001).

**TABLE 2:** Logistic regression for melanoma specific mortality in Michigan 2014–2018

Variable	Risk ratio	95% CI	*p* value
Gender (Male)	2.213	(1.94, 2.52)	<.001
Age (≥65)	3.986	(3.52, 4.53)	<.001
Area of residence (rural)	1.491	(1.27, 1.74)	<.001

*Note*: Covariates are gender, age and area of residence.

Our physician supply search resulted in 463 dermatologists in urban Michigan counties for a ratio of 17 687 persons/dermatologist, while rural counties have 51 dermatologists for a ratio of 35 249 persons/dermatologist. There was no significant difference in the incidence rates for CM in rural counties with or without a dermatologist (22.94/100 000 persons vs. 23.45/100 000 persons, *p* = .53). Seven of the 10 rural counties without a dermatologist with the highest incidence rates for CM had neighboring counties with a dermatologist and all 10 of the rural counties without a dermatologist and the lowest incidence rates of CM bordered a county with a dermatologist.

## 4 DISCUSSION

Our findings identified significant differences in the incidence rates of CM and the melanoma‐specific mortality in rural Michigan counties when compared with those residing in urban counties. The rural–urban disparities in incidence rate increased with each subsequent advance in SEER summary stage of melanoma (Table 1). Rural–urban disparities in incidence of CM in Michigan from 2014–2018 were seen in significant differences for these stages: Localized (RR = 1.22; 95% CI, 1.16–1.29; *p <* .001); Regional (RR = 1.38; 95% CI, 1.20–1.59; *p* < .001); and Distant (RR = 1.54; 95% CI, 1.28–1.85; *p* = .001). Incidence was also higher for all Michigan citizens, both age groups and both genders in our study. Most concerning was the significant higher melanoma‐specific mortality in rural Michigan from 2014–2018. The higher disease‐specific mortality was seen in all patients, both age groups, both genders and in logistic regression when controlled for age and gender (Table 1). This disparity is best summarized by the RR of 1.55 (95% CI = 1.37, 1.75; *p* = .001) for rural–urban melanoma‐specific mortality.

Our study did identify some potential reasons for the disparities we found. We hypothesized a lack of dermatologists in rural counties may correlate with the disparities we found. Michigan has almost twice the number of dermatologists per capita practicing in its urban counties (17 687 persons/dermatologist verses 35 249 persons/dermatologist). Only 22/62 rural Michigan counties have a practicing dermatologist within their boundaries. Despite these findings there was no significant difference in overall incidence rate of CM in rural Michigan counties with verses without a dermatologist, respectively (22.94/100 000 persons vs. 23.45/100 000 persons, *p* = .53). This last observation is not consistent with a 2007 study, which found that the lack of a local dermatologist and the farther the distance between patients and a dermatologist correlated with a higher stage of melanoma at the time of diagnosis.^13^ The significantly higher incidence of localized stage CM argues against lack of early detection in rural Michigan patients. Given that 92.4% of US melanoma occurs in patients identified as white, racial differences may account for our disparate mortality. Due to suppressed data for race from both the CDC and the Michigan Department of Health and Human Services, we were unable to collect data for any racial group in rural Michigan other than white.^11^ Given the 21% higher white population in rural compared with urban Michigan, race could account for some of the disparities we identified.^3^ Rural residents may have greater ultraviolet light exposure; however, there was only a slight increase in the percentage of rural versus urban residents working in agricultural, hunting, or mining occupations (3.5% vs. 0.6%).^3^ Additionally, income may influence the mortality in rural areas. Affluence has been shown to have a survival benefit for CM in Scotland.^7^ According to the 2017 United States Department of Agriculture Economic Research Service report, the per capita income is 17.9% lower in rural versus urban Michigan.^14^

Limitations of our study include the inability to use race as a covariate due to suppression of data for all racial groups in rural Michigan other the white due to concerns for individual patient identification. Therefore, Race represents a possible cofounder. Due to incomplete data on insurance status, we were unable to use these data as a covariate. Urban and rural counties were each used as a statistical entity, the health of patients within each county likely represents a spectrum rather than a statistical representation. Lastly, the data involves only one US State with regional significance over a 5‐year period.

## 5 CONCLUSION

Cutaneous melanoma has a higher incidence and age specific mortality rate in rural areas compared to urban areas of Michigan. Increasing the awareness of various disparities such as race, occupation, and the supply of dermatologists is critical for early detection of melanoma and access to treatment. Melanoma presently is the third most common cancer afflicting both men and women in the rural United States. Our findings highlight the dearth of care and resources and demonstrate its connection to patient outcomes, which are important to encourage the field to prioritize medical care in rural areas.

## AUTHOR CONTRIBUTIONS

**Richard A. Shellenberger:** Conceptualization (lead); data curation (lead); formal analysis (lead); investigation (lead); methodology (lead); project administration (lead); resources (lead); software (lead); supervision (lead); writing – original draft (lead); writing – review and editing (lead). **Timothy M. Johnson:** Conceptualization (supporting); data curation (supporting); formal analysis (supporting); methodology (supporting); writing – original draft (supporting); writing – review and editing (supporting). **Fatima Fayyaz:** Conceptualization (supporting); data curation (equal); formal analysis (supporting); visualization (supporting); writing – original draft (supporting). **Bhanu Swamy:** Conceptualization (supporting); data curation (supporting); formal analysis (supporting); software (supporting); visualization (supporting); writing – original draft (supporting); writing – review and editing (supporting). **Alan C. Geller:** Conceptualization (equal); data curation (supporting); formal analysis (supporting); funding acquisition (equal); methodology (equal); writing – original draft (equal).

## CONFLICT OF INTEREST

The authors have stated explicitly that there are no conflicts of interest in connection with this article.

## Data Availability

All the data to support the findings of this study are reported in Tables 1 and 2 were calculated by the authors of this study and were based on data obtained from the Centers for Disease Control and Prevention (U.S. Cancer Statistics Data Visualizations Tool | CDC) and the Michigan Resident Cancer Incidence File, Michigan Department of Health and Human Services, Division of Vital Records and Health Statistics (Vital Statistics (michigan.gov)). These data are available on request from the corresponding author and available to the public.
